# Exploring Adenosine Receptor Ligands: Potential Role in the Treatment of Cardiovascular Diseases

**DOI:** 10.3390/molecules22060917

**Published:** 2017-06-01

**Authors:** Werner J. Geldenhuys, Ahmad Hanif, June Yun, Mohammed A. Nayeem

**Affiliations:** 1 Department of Pharmaceutical Sciences, West Virginia University, School of Pharmacy, Morgantown, WV 26506, USA; werner.geldenhuys@hsc.wvu.edu (W.J.G.); ahanif@hsc.wvu.edu (A.H.); 2 Department of Integrative Medical Sciences, Northeast Ohio Medical University College of Medicine, Rootstown, OH 44272, USA; Jyun@neomed.edu

**Keywords:** atherosclerosis, myocardial infarction, cardiac death, vascular tone

## Abstract

Cardiovascular diseases remain the number one diseases affecting patients’ morbidity and mortality. The adenosine receptors are G-protein coupled receptors which have been of interest for drugs target for the treatment of multiple diseases ranging from cardiovascular to neurological. Adenosine receptors have been connected to several biological pathways affecting the physiology and pathology of the cardiovascular system. In this review, we will cover the different adenosine receptor ligands that have been identified to interact with adenosine receptors and affect the vascular system. These ligands will be evaluated from clinical as well as medicinal chemistry perspectives with more emphasis on how structural changes in structure translate into ligand potency and efficacy. Adenosine receptors represent a novel therapeutic target for development of treatment options treating a wide variety of diseases, including vascular disease and obesity.

## 1. Introduction

The cardiovascular system plays an important role in the health and well-being of patients. Dysregulation of the cardiovascular system has large implications, for example hypertension can be a major risk factor for the development of stroke and heart disease. It has been found that more than 30% of adults will run the risk of dying from either heart disease or stroke [1]. This has led to an increased search for the understanding of the pathology behind these life-threatening disease states, and is evident in both medical literature as well as in the health care commercial market.

The vascular system plays an important role in the normal physiology, affecting several organ systems, including the brain and renal system. Any pathological changes in this system can lead to chronic disease states, including altering the structure of blood vessels in the kidney and may lead to cerebrovascular diseases such as Alzheimer’s disease. In the clinical setting, patients who are being treated for cardiovascular diseases, easily experience polypharmacy as the normal treatment paradigm. Although several classical drug targets exist in the treatment of the cardiovascular system, e.g., beta-antagonists, voltage gated calcium blockers (VGCC) etc., a deeper knowledge is needed to functionally treat this system to achieve the maximal decrease in mortality and morbidity. One of the newer drug targets which we are investigating is the adenosine receptor signaling cascade and its effect on cardiovascular physiology.

## 2. Role of Adenosine in Cardiovascular Function

Adenosine is a purine nucleoside which plays an important physiological role due to the presence of the adenosine receptors in practically every tissue type [2,3]. There are four distinct adenosine receptors, which are known as A_1_, A_2A_, A_2B_, and A_3_. The usefulness of adenosine, physiologically, can be seen from the fact that adenosine, itself, is used clinically (as an injectable generic product), for the treatment of supraventricular tachycardia [4].

The adenosine receptors belong to the G protein-coupled receptors (GPCRs) family of transduction receptors. These are functionally arranged in a seven-membrane spanning α-helical system which is known as seven transmembrane receptors (7TMRs) [5]. The adenosine receptors A_1_, A_2A_ and A_2B_ have been shown to play important roles in the peripheral cardiovascular system; for instance, A_1_ and A_2B_ play a role in the vasomotor response of the mesenteric artery, and play a role in lipolysis from adipose tissue, as well as other diverse effects including inflammation and oxidative stress [6,7,8].

**Figure 1:**
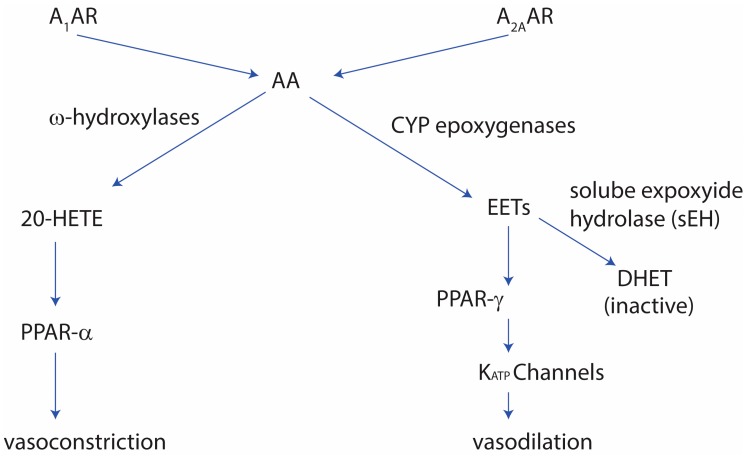
The role of adenosine receptors in vascular tone regulation. See text for details. A_1_AR: adenosine A_1_ receptor; A_2A_AR: adenonsine A_2A_ receptor; AA: arachidonic acid; 20-HETE: 20-hydroxyeicosatetraenoic acid; EETs: epoxyeicosatrienoic acids; and DHETs: dihydroxyeicosatrienoic acids.

Adenosine A_1_ receptor couples to Gα_i_ (1–3) and Gα_o_ receptors, and currently is thought to be the main signaling mechanism that leads to a decrease in intracellular cAMP, and activates the RISK kinase ERK1/2 [5,9]. Alternatively, the adenosine A_1_ receptor can also signal via interaction with the b-arrestins, which can also lead to the activation of ERK1/2 kinases [10]. See Figure 1 for a schematic overview of the adenosine receptor signaling cascade. The adenosine A_2A_ receptor couples to Gαs and the G_olf_ proteins and stimulation of the receptor leads to the accumulation of cAMP, as well as mediating the activation of ERK1/2 [11,12].

Adenosine receptors play an important role in the physiology of the cardiovascular system. For instance, in coronary smooth muscle cells (CSMC), the activation of adenosine A_1_ receptors can be protective against ischemic events in cell culture. The stimulation of the adenosine A_1_ receptor in these cells was found to upregulate iNOS, HSP27, as well as PKC-epsilon signaling in the CSMC. Additional studies indicated that the activation of the adenosine A_1_ receptors stimulate the PKC kinases alpha, beta, gamma, epsilon and zeta, but not delta and mu. Overexpression of adenosine A_1_ receptors was shown to offer protection against ischemic stress, primarily via the iNOS and K_ATP_ channel interaction [13,14,15,16].

Adenosine A_2A_ receptors play a role in vascular tone where they cause vasodilation. This vasodilatation activity is thought to be mediated by the cytochrome P-450 cyp epoxygenases, and that the knockout of the adenosine A_2A_ receptor leads to vasoconstriction via the CYP4a cytochrome P-450 system. Further studies showed that the role adenosine A_2A_ receptor plays in vasodilation is thought to help in the avoidance of salt sensitivity, and that under high salt conditions, adenosine A_2A_ receptor leads to increased expression of the CYP2C29 epoxygenases. The effect of high salt concentrations in the diet leads to increase in blood pressure. In mice fed a high-salt diet, there was an increased expression of A_2A_ receptors and CYP2J2, whereas the expression of soluble epoxide hydrolase (sEH), CYP4a, and adenosine A_1_ receptors was decreased. The latter signaling of adenosine A_1_ receptor causes vasoconstriction in the normal-salt diet. The vasodilation by adenosine A_2A_ receptors via the CYP epoxygenases was shown to persist in mice where endothelium NOS (eNOS) was knocked out, and the adenosine A_1_ receptor-induced vasoconstriction via sEH and CYP4A was persistent, indicating the constitutive pathway is not dependent on the NO signaling [17,18,19,20].

The role of adenosine A_2A_ receptors in the vasodilation activity was shown to be linked to the MAP-kinases In mice, where the adenosine A_2A_ receptor was knocked out, mice showed increased contraction when exposed to adenosine The increased vasoconstriction was thought to occur via an upregulation of adenosine A_1_ receptors and CYP4a can lead to upregulation of PKC-alpha which can activate the PKC-alpha-ERK1/2 pathway [20,21]. Additionally, the adenosine A_2A_-mediated vasodilation was found to relate to soluble expoxide hydrolase (sEH). In sEH-knockout mice, the mice showed an increase in adenosine A_2A_ receptor expression, as well as CYP2J and PPAR-gamma, whereas the adenosine A_1_ receptor was decreased, along with PPAR-alpha [22,23]. The high-salt diet can augment the vascular contraction in adenosine A_2A_-knockout mice, due to the increased adenosine A_1_ receptor levels. Normally adenosine A_2A_ receptor shows enhanced vasodilation in a high-salt diet due to increased cyp-expoxygenases-derived epoxyeicosatrienoic acids (EETs) in the vascular system [23], as well as PPAR-gamma and K_ATP_ channels [24], PPAR-gamma, and K_ATP_ channels. Furthermore, in sEH-overexpressed mice, adenosine A_1_ receptor levels are increased, vascular tone is increased, and K_ATP_ channel-mediated relaxation is decreased [24]. Figure 1 summarizes the role adenosine receptors play in the vascular tone.

## 3. Medicinal Chemistry

The adenosine receptor family has been targeted by several groups to discover novel compounds which can modulate the function of these receptors [5,25]. One major breakthrough in this field was the crystallization of the adenosine A_2A_ receptor by the group of Jaakola et al. [26].

**Figure 2:**
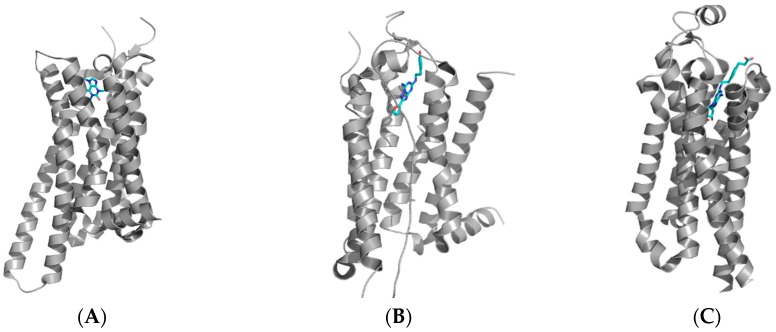
Structures of the adenosine A_2A_ receptor with (**A**) caffeine (PDB: 3RFM); (**B**) the antagonist ZM241385 (PDB: 3EML); and (**C**) the agonist CGS21680 (PDB: 4UG2). It has been shown that the extracellular loops play an important role in the agonist/antagonist interaction of compounds with the receptors. These crystal structures have been used to design novel small organic compounds which can be used to target the adenosine receptors.

**Figure 3:**
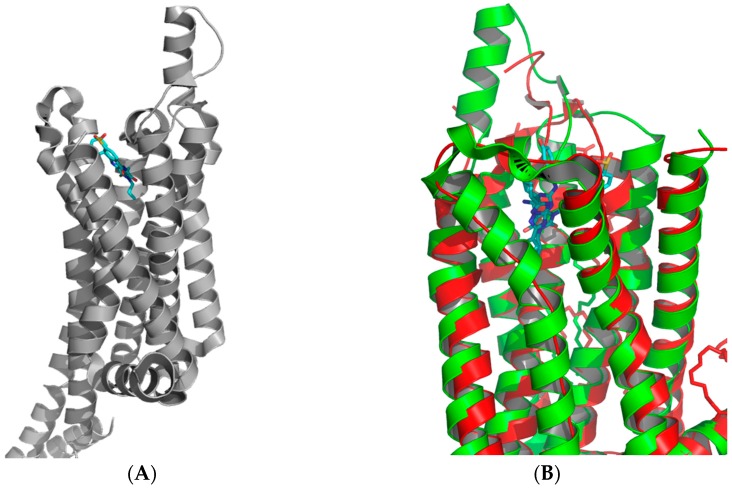
Structure of (**A**) adenosine A_1_ receptor bound with the covalent antagonist DU172 (PDB: 5UEN); (**B**) overlay of the A_1_ (green) and A_2A_ (red) receptors showing the significant difference in the extracellular loop region (ECL) between the two receptors [26,27].

Table 1 shows the different adenosine receptors which have been crystallized and the protein data bank access codes (PDB). GPCRs and transmembrane receptors were notoriously difficult to crystallize before the adenosine A_2A_ receptor crystal was solved. The subtype selective antagonist ZM241385 was co-crystalized and was shown to bind in a manner which changed the interaction between the helical loops and the internal core of the protein (PDB file 3EML). Of note with GPCRs is the extracellular loops (ECL), which have been shown to play an important role in the GPCR function, with noted specific structural changes with agonist and antagonist interactions. Table 1 shows the deposited Protein Data Bank codes for the selected different crystal structures of the adenosine A_2A_ receptor, as well as adenosine A_1_ receptor. Recently, the adenosine A_1_ receptor was crystallized by the group of Glukhova et al. [27] and revealed an interesting difference in the binding pocket between the adenosine A_2A_ and A_1_ receptors. A few noticeable differences include the ECL, which was significantly different in the adenosine A_1_ receptor, and the presence of a wider extracellular binding cavity for ligands. Additionally, a secondary binding pocket of the primary binding pocket of adenosine A_1_ receptor was found, when compared to the A_2A_ receptor (Figure 2 and Figure 3).

For adenosine A_3_ receptor, citations for the homology models are shown, but to this date there has not been a crystal structure solved for this isoform. For instance, the group of Almerico et al. [28] used adenosine A_2A_ receptor structure in conjunction with homology modeling to develop a homology model of adenosine A_3_ receptor. This model was then used to mine the ZINC database for novel adenosine A_3_ receptor ligands, using a combination of docking and quantitative structure-activity relationships (QSAR) techniques.

**Figure 4:**
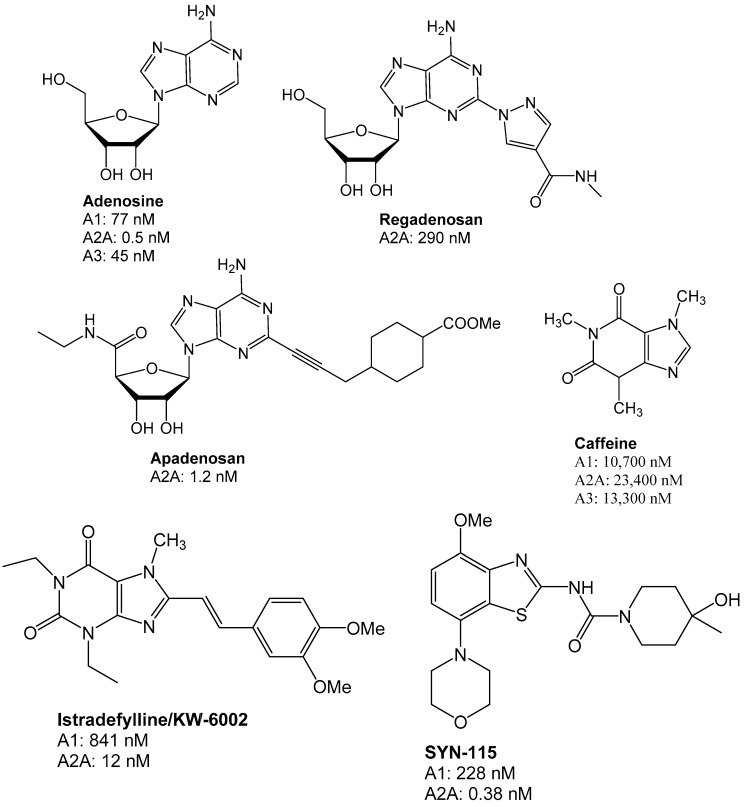
Structures of adenosine ligands which were evaluated in clinical trials. The therapeutic areas represented a range from cardiovascular to neurodegenerative diseases such as Parkinson’s disease [25]. For a more in-depth review, the reader is referred to a recent review [3].

The adenosine receptor has been targeted in the past few years for therapeutic drug development, as can be seen by the number of clinical trials featuring these ligands. Figure 4 shows several of the compounds which have been evaluated. The reader is referred to an excellent review on the clinical trials of these compounds [3]. The therapeutic areas targeted by these clinical trials range from cardiovascular to neurodegenerative diseases, such as Parkinson’s disease. For instance, for several years the adenosine A_2A_ receptor antagonist istradefylline (KW-6002, a styrylxanthine) has been evaluated for clinical efficacy in Parkinson’s disease. Although istradefylline did not get approval by the USA FDA, it was recently approved for use in Japan for the use in patients [29]. Adenosine has been on the market for a few years in an injectable form e.g., Teva’s Adenoscan^®^, while caffeine is available in several generic forms to treat sleep apnea of prematurity in infants.

**Figure 5:**
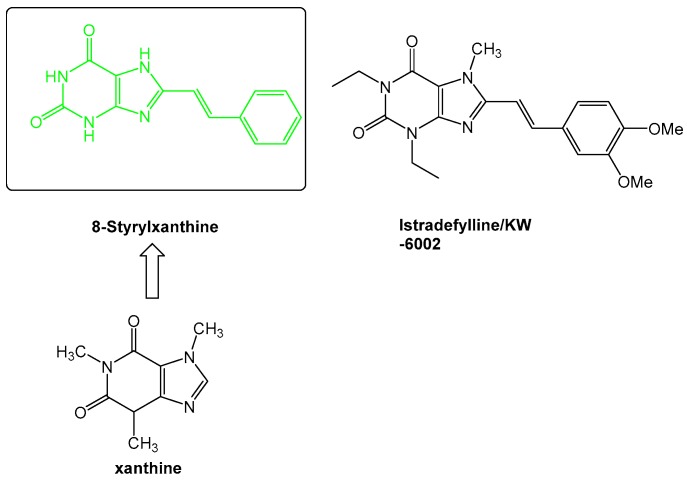
The progression from xanthines, such as caffeine, to the novel compound istradefylline, which was recently approved for the use in Parkinson’s diseases.

The medicinal chemistry surrounding the development of novel adenosine receptor ligands was largely driven in the beginning by derivatization of the adenosine scaffold to gain understanding of the structure-activity relationships especially between adenosine A_1_ and A_2A_ receptors. From these studies, novel scaffolds were developed, such as the core styrylxanthine from which istradyfilline (KW-6002) was derived (Figure 5).

**Figure 6:**
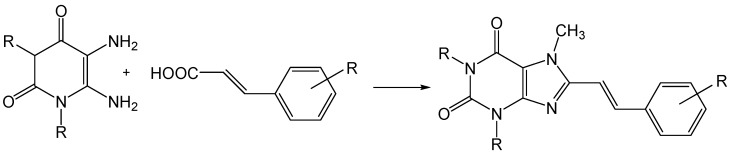
Synthesis route of styrylxanthines such as istradyfilline.

Compounds such as the 8-styrylxanthines, of which istradyfilline is a member, can easily be prepared by the acylation of a diaminouracil and a trans-cinnamic acid (Figure 6) [30].

**Figure 7:**
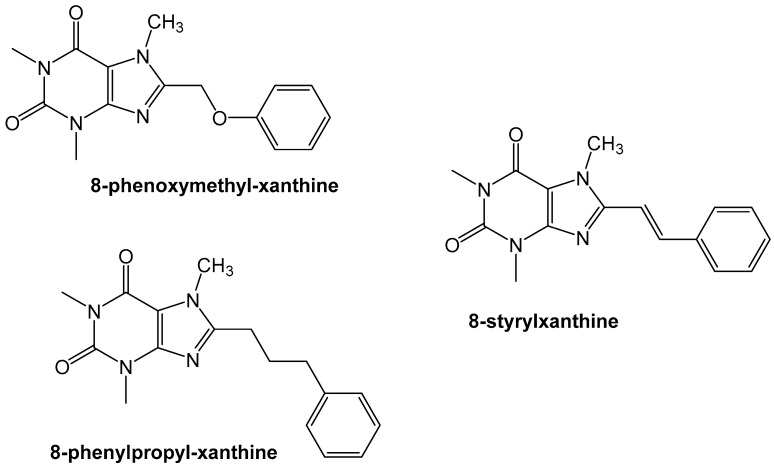
The styrylxanthine scaffold is preferred for A_2A_ receptor binding when compared to the phenoxymethyl or phenylpropyl xanthines [31].

Exploration of the adenosine A_2A_ receptor antagonism led to the group of Van der Walt et al. to evaluate the effect of the styryl-moiety. They found that the styryl moiety was important for developing high affinity compounds (~Ki < 100 nM) whereas the phenoxymethylxanthine and phenylpropylxanthines did not show similar high affinity binding to the A_2A_ receptor (~Ki > 0.5 nM) (Figure 7) [31].

**Figure 8:**
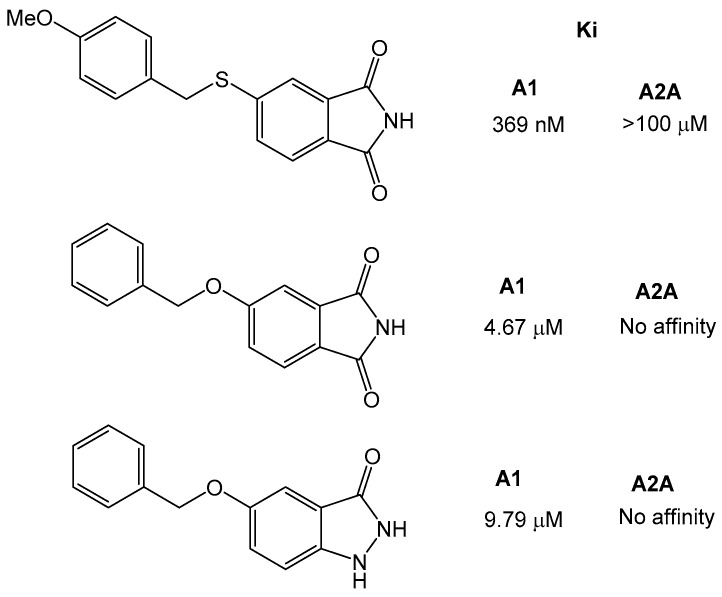
Structures of the phthalimide and istatin based compounds and their affinity for the adenosine receptors [32].

Further studies also showed that the xanthine moiety can be replaced and still retains adenosine receptor activity [32]. The group of Van der Walt et al., synthesized a series of sulfanylphthalimide analogues and tested them for adenosine A_1_ and A_2A_ receptor affinity. The phthalimide moiety has been found in other drug-like compounds, such as thalidomide or pomidomide. The results showed that the compounds were selective for adenosine A_1_ over A_2A_ receptor, and that the most potent compound was 5-[(4-methoxybenzyl) sulfanyl]phthalimide with a KI of 369 nM. Similarly, the 5-benzylopxyphatalimide and 5-benzyloxyistatin were inactive against the A_2A_ receptor, and the affinity for A_1_ was lower (Ki ~5 µM) than for the sulfanyl-derivatives (Figure 8).

**Figure 9:**
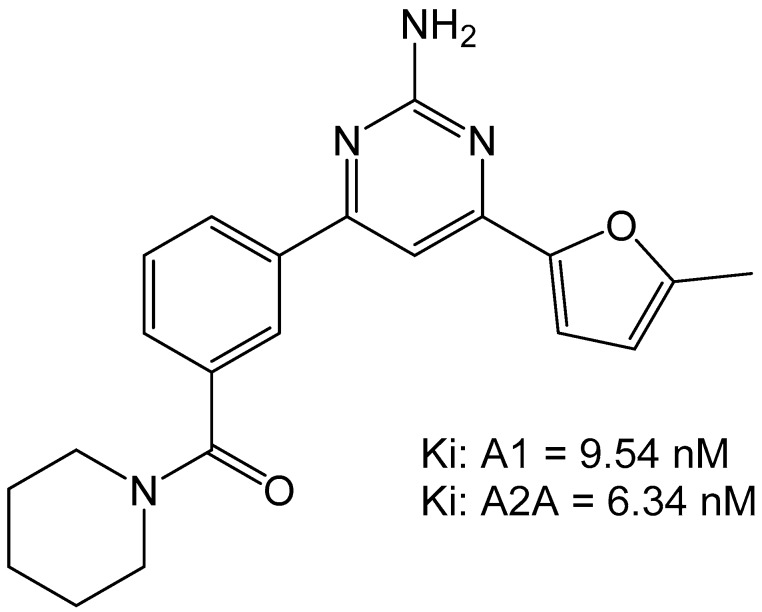
Structure of the 2-amiopyridine that was found to be a dual A_1_/A_2A_ receptor [33].

The group of Robinson et al., characterized a small set of 2-aminopyridines as dual A_1_/A_2A_ antagonists. These compounds were thought to have utility in motor diseases such as Parkinson’s disease but would have potential for use in the cardiovascular system as well. The most potent compound found from this campaign was 4-(5-Methylfuran-2-yl)-6-[3-(piperidine-1-carbonyl)phenyl]pyrimidin-2-amine which was able to bind to A_1_ with a Ki of 9.54 nM and to the A_2A_ with an Ki of 6.34 nM (Figure 9). The compound did not show any toxic activity in vitro and was able to attenuate haloperidol-induced catalepsy in rats when dosed at 1 mg/kg. This suggests that the compound would be amenable to future development for in vivo use [33].

**Figure 10:**
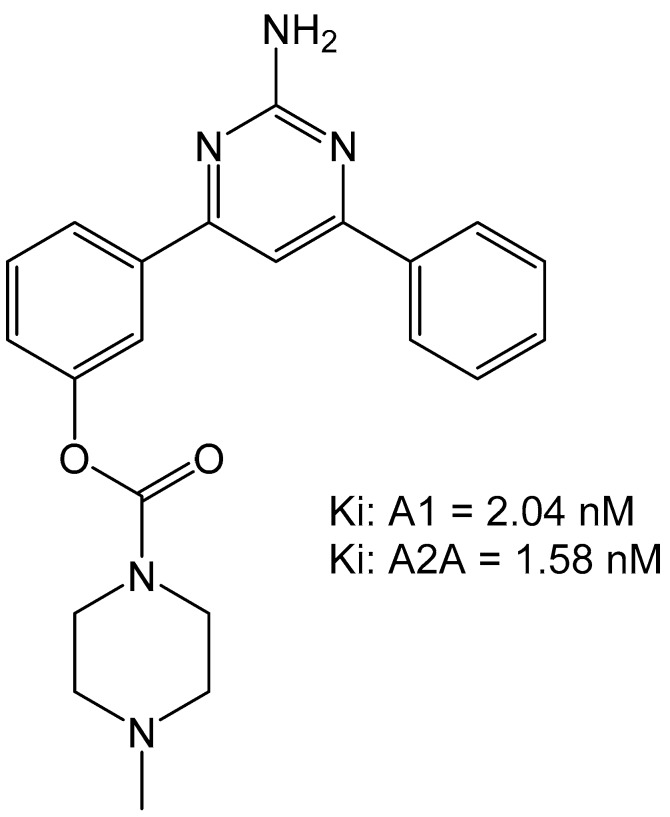
Carbamate-based adenosine receptor antagonists [34].

Carbamate-pyrimidines were prepared to evaluate their binding to the adenosine A_1_ over A_2A_ receptors due to the carbamate moiety featuring in several drug-types. The group of Robinson et al., prepared a set of carbamate compounds and found that 3-(2-amino-6-phenylpyrimidin-4-yl)phenyl morpholine-4-carboxylate compound was the most potent compound with an Ki of 2.65 nM for adenosine A_1_ receptor and an Ki of 3.50 nM for the adenosine A_2A_ receptor (Figure 10) [34]. This compound was also able to reverse catalepsy in rats when dosed at 0.4 mg/kg, suggesting effective in vivo therapeutic potential.

**Figure 11:**
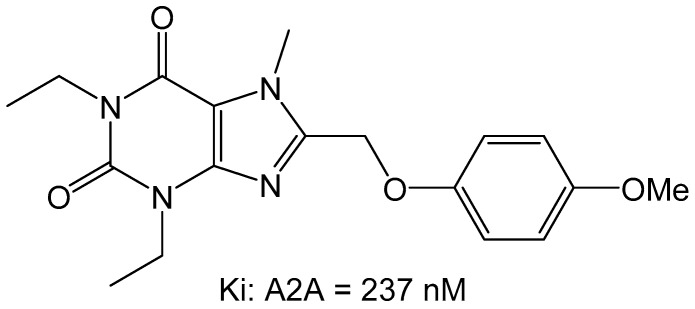
Phenoxymethyl-xanthine derivatives which are dual A_1_/A_2A_ antagonists [35].

Since previous studies have suggested that the styrylxanthine can be used to develop dual adenosine A_1_ and A_2A_ receptor ligands, the group of Harmse et al., synthesized a series of *para*-substituted 1,3-diethyl-7-methyl-8-(phenoxymethyl)-xanthine analogs [35]. They found that para substitution on the phenoxymethyl side chain increased the affinity for the adenosine A_2A_ receptor, with methoxy (OCH3) being the most potent with a Ki of 237 nM (Figure 11).

Additionally, it was found that this compound behaved as an antagonist when screened in the GTP-shift assay using rat brain membranes.

**Figure 12:**
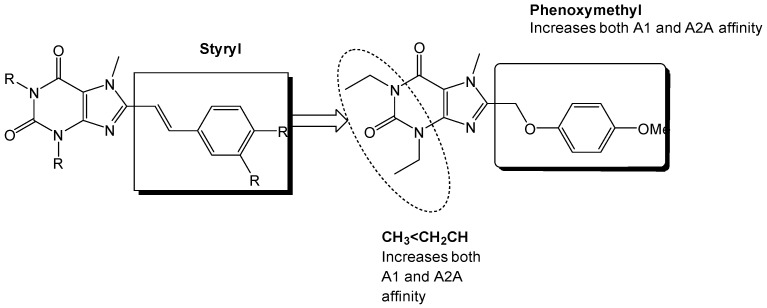
Substitution on the styryl moiety with the phenoxymethyl side chain leads to dual A_1_/A_2A_ receptor antagonists [35].

**Figure 13:**
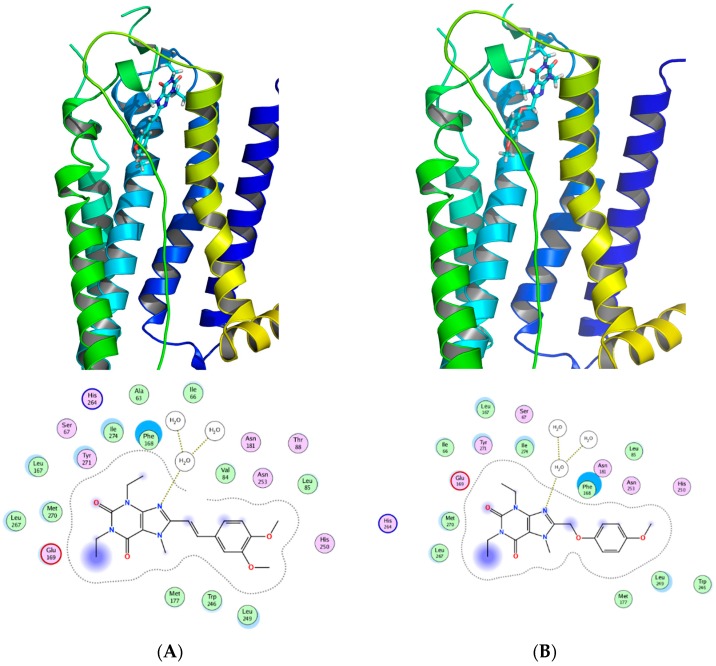
Docking of (**A**) istradyfilline (KW-6002) and the (**B**) phenoxymethyl-xanthine derivative in the adenosine A_2A_ receptor (3EML). The two compounds share a binding motif with the coordination with the water in the binding pocket of adenosine A_2A_ receptor.

Figure 12 shows a SAR synopsis from the studies of the phenoxymethyl replacement of the styryl moiety. We employed docking studies to investigate the differences between the two compounds in their affinity for the adenosine A_2A_ receptor (Figure 13), using MOE 2016 [36]. We found that both compounds orient with the xanthine pointed to the outside of the pocket, and the side chain oriented inwards. A crystal water molecule seems to be bridging the compounds with hydrogen bonds and allows for interaction with the protein.

## 4. Conclusions

The adenosine receptor system plays an important physiological role in the cardiovascular response. Considering the signaling role they play, these receptors represent a novel class of drug targets which can have phenotypic and therapeutic potential to treat people with high blood pressure and related cardiovascular disorders. As we gain understanding in the mechanism of action of these receptors, we will undoubtedly be able to develop effective adenosine ligands for use in cardiovascular diseases.

## Figures and Tables

**Table 1:** Crystal structures of the adenosine receptors which can be used for drug discovery projects.

A_1_	A_2A_
5UEN	3EML
	3RFM
	2YDO
	2YDV
